# Reliability and Validity of the Interactive Drawing Test: A Measure of Reciprocity for Children and Adolescents with Autism Spectrum Disorder

**DOI:** 10.1007/s10803-014-2353-x

**Published:** 2015-01-29

**Authors:** Tineke Backer van Ommeren, Hans M. Koot, Anke M. Scheeren, Sander Begeer

**Affiliations:** 1 Department of Developmental Psychology, VU University Amsterdam, Van der Boechorststraat 1, 1081 BT Amsterdam, The Netherlands; 2 EMGO Institute for Health and Care Research, VU University, Amsterdam, The Netherlands; 3 School of Psychology Australia, University of Sydney, Sydney, Australia; 4 Amsterdam Brain and Cognition, University of Amsterdam, Amsterdam, The Netherlands

**Keywords:** Autism spectrum disorders, Assessment, Social behavior, Validity, Reliability

## Abstract

Poor reciprocity is a defining feature of an autism spectrum disorder (ASD). In the current study, we examined the reliability and validity of the Interactive Drawing Test (IDT), a new instrument to assess reciprocal behavior. The IDT was administered to children and adolescents with ASD (n = 131) and to a typically developing group (n = 62). The IDT had excellent inter-rater reliability and moderate to good test–retest reliability. The results showed clearly distinctive response patterns in the ASD group compared to the typically developing group, independent of verbal IQ and age. Convergent validity of the IDT was low. Sensitivity and the predictive accuracy of the IDT for detailed levels of reciprocal behavior in autism are discussed.

## Introduction

Poor reciprocity is a defining feature of an autism spectrum disorder (ASD). Reciprocity is defined as the participation in a dynamic process of mutual, equal or complementary interaction and sharing with another person (Gallagher 2004; Gernsbacher 2006; Komorita et al. 1992; Trevarthen and Aitken 2001). The DSM-5 includes deficits in reciprocity as a *necessary* criterion for an ASD diagnosis, whereas the DSM-IV included it as a *possible* criterion (APA 2000, 2013). The DSM-5 specifies the deficits in reciprocity of individuals with ASD as “ranging from abnormal social approach and failure of normal back-and-forth conversation; to reduced sharing of interests, emotions, or affect; to failure to initiate or respond to social interactions” (APA 2013, p. 50). Despite the central role of reciprocal behavior in the definition and diagnostic criteria of autism, direct measures of real life reciprocity are rare. The Interactive Drawing Task (IDT) is, to our knowledge, the first instrument designed to specifically assess the level and quality of reciprocal behavior. This study tests the reliability and validity of the IDT in a large sample of children and adolescents with and without ASD.

When relying on currently available clinical assessment tools, reciprocal behavior of children with ASD can be measured based on specific elements of parent reports or clinician observations. For instance, some items of parent interviews (e.g., the Autism Diagnostic Interview-Revised, ADI-R; Rutter et al. 2006) and questionnaires (e.g., the Social Responsiveness Scale, SRS; Constantino and Gruber 2005, the Social Communication Questionnaire, SCQ; Rutter et al. 2003) include descriptions of reciprocal behavior, e.g., “your child avoids initiating social interaction with peers or adults” or “your child is awkward in turn taking during interactions” (SRS). In addition, several other surveying assessment tools provide proxies for social reciprocity, such as the Childhood Autism Rating Scale second edition, CARS-2 (Schopler et al. 2010, the Modified Checklist for Autism in Toddlers, M-CHAT (Robins et al. 2001). Clinicians generally rely on the Autism Diagnostic Observation Schedule (ADOS; Lord et al. 2000) to assess reciprocal behaviors. The ADOS is designed to create multiple and diverse opportunities for reciprocal interactions, which can be observed and rated. The ADOS and other current instruments thus provide extensive information about vital social skills for reciprocity and include, among other domains, the *presence* of reciprocal behavior as outcome measure. The IDT was developed to add to these assessment tools for reciprocity by providing not only a specific assessment of the *presence* of reciprocal behavior and but also an assessment of the *quality* of reciprocal behavior.

The IDT is an interactive test procedure designed to elicit reciprocal behavior. The test relies on the detailed coding of the behaviors of two people (the participant and the researcher) during the creation of a mutual drawing. Reciprocal behavior (“reciprocal drawing”) during the interaction process is reflected by the relative frequency that participants contribute meaningful elements to a mutual drawing object. For instance, one participant may add apples to a drawing of a tree, and the other participant may draw a ladder. The joint contribution of both participants to meaningful objects in the drawing is referred to with reciprocal drawing.

The test also registers who initiates a drawing object. A pilot study indicated that participants with ASD showed particularly low reciprocity when objects were initiated by the researcher (Backer van Ommeren et al. 2012a). For example, when the researcher initiated an object (e.g., a car), participants with ASD were less inclined to contribute to the researcher’s object (e.g., by drawing a steering wheel in the car). This is referred to as “reciprocity in other’s initiative”. In addition, the IDT codes reciprocal turn taking behavior and flexibility in response to additions by the researcher to the participants’ own drawing objects, providing a full perspective on the reciprocal interaction.

The IDT is suitable for a wide age range, from 6 years old up to adulthood, and requires minimal verbal skills. Children with a developmental age of 6 years likely have required enough social skills and fine motor skills needed for an adequate IDT performance. Despite strong evidence for its sensitivity (Backer van Ommeren et al. 2012a, b), the pilot study lacked essential information. Test–retest reliability of the IDT was not analyzed. Convergent validity was assessed only by comparison with parent reported autistic traits [the Social Responsiveness Scale (SRS; Constantino and Gruber 2005), but not by using structured observations by clinicians (i.e., ADOS scores]. Furthermore, the pilot study involved a relatively small sample (total n = 49), and lacked specific age groups (early adolescents).

The goal of the present study is to test the reliability and validity of the IDT in a large sample of children and adolescents with and without ASD (n = 193), aged 6–18 years. Inter-rater reliability was tested within the main sample, test–retest reliability was determined in a separate sample (n = 29, including 14 participants with ASD and 15 TD participants). We addressed criterion validity by analyzing differences in IDT performance between participants with ASD and typically developing participants in the main sample. Convergent validity was addressed by analyzing the relation between IDT scores and standardized diagnostic instruments for autism, the SRS and the ADOS. Divergent validity was explored by testing the association of IDT scores with language ability, age, and gender of the participants.

## Method

### Participants of Main Study

**Fig. 1:**
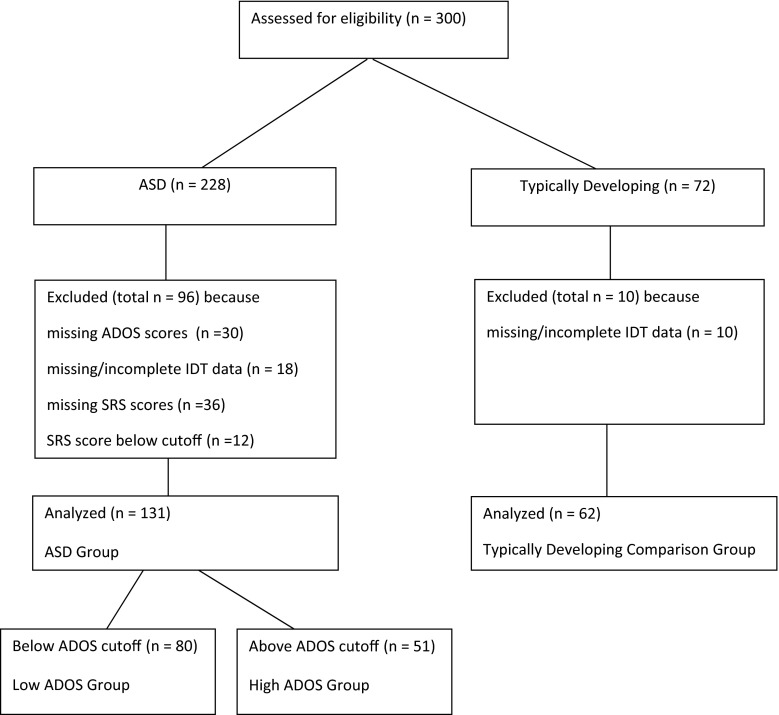
Flow of participants

Participants of the main study included 193 children and adolescents, 131 with ASD (114 boys, 17 girls; mean age 13.4 years, SD 3.0 years) and 62 with typical development (55 boys, 7 girls; mean age 12.3 years, SD 2.8 years) (see Table 1 for details, and Fig. 1 for an overview of the flow of participants). Children and adolescents with ASD were recruited from special primary and secondary schools in the Amsterdam region, who offer education for children with autism without intellectual impairments. Children were included based on a clinical diagnosis established prior to recruitment according to DSM-IV-TR criteria (APA 2000) by an independent team of clinicians, including psychiatrists and/or psychologists. They were not involved in the current research project. The diagnostic process included parent interviews, psychiatric examinations of the child, school observations and neuropsychological testing. The clinical diagnoses of the 131 ASD participants were all confirmed by clinically elevated scores on the Social Responsiveness Scale (Constantino and Gruber 2005), which was administered as part of the current study. The comparison group was recruited via public primary and secondary schools in the Amsterdam region.

**Table 1:** Descriptives for the High ADOS group, the Low ADOS group, the total ASD group and the typically developing comparison group

Child variables	High ADOS group (n = 51)		Low ADOS group (n = 80)		Total ASD group (n = 131)		Comparison group (n = 62)		Group differences
	M (SD)	Range	M (SD)	Range	M (SD)	Range	M (SD)	Range	
Age (in years)	12.7 (2.9)	6.4–18.4	13.8 (3.0)	6.9–18.7	13.4 (3.0)	6.4–18.7	12.3 (2.8)	6.0–17.8	(High < Low) & ASD > C**
Receptive vocabulary skills (PPVT)	103 (13.6)	64–126	107 (13.8)	66–132	105 (13.8)	64–132	106 (12.9)	77–132	n.s.
Gender (n boys; girls)	49; 2		65; 15		114; 17		55; 7		Boys: High > Low*
Total ADOS (3 or 4)	10.6 (3.1)	7–19	3.4 (1.9)	0–6	6.5 (4.4)	0–19	–	–	High > Low, High > ASD**
Total SRS	85.8 (17.7)	60–82	87.3 (16.9)	61–133	86.7 (17.2)	60–133	34.5 (19.4)	13–111	High & Low & ASD > C**
Educational level mother^a^	5 (1.7)	1–7	4.9 (1.7)	1–7	4.9 (1.7)	1–7	5.4 (1.8)	2–7	n.s
Educational level father^a^	4.5 (1.9)	1–7	4.8 (1.6)	1–7	4.7 (1.7)	1–7	5.2 (1.6)	2–7	High < C*
Level profession mother^b^	2.1 (2.1)	0–5	2.0 (1.9)	0–5	2.0 (2.0)	0–5	1.9 (1.8)	0–5	n.s
Level profession father^b^	1.7 (1.7)	0–5	1.6 (1.5)	1–5	1.6 (1.5)	1–5	1.6 (1.5)	2–5	n.s
Number living with both biological parents	84 %		75 %		79 %		63 %		n.s.

* *p* < .05; ** *p* < .01

^a^1 = elementary school; 2 = intermediate vocational education; 3 = middle general secondary education; 4 = middle vocational education; 5 = higher general secondary education; 6 = higher vocational education; 7 = academic education

^b^0 = no profession; 1 = elementary; 2 = lower; 3 = middle; 4 = higher; 5 = academic level

In addition to the SRS, we also administered the ADOS as part of the current study. Despite their clinical diagnoses, admission to specialized education centers for autism, and parental reports of autistic traits above the clinical threshold on the SRS (*M* = 86.74, *SD* = 17.20), the average ADOS score of the 131 ASD participants (*M* = 6.47, *SD* = 4.35) indicated that a majority of the ASD sample (61 %; n = 80) received an ADOS score below the ASD cutoff (<7) using the revised ADOS algorithm (Gotham et al. 2009). To ensure that any possible group difference between participants with ASD and typically developing (TD) participants on the IDT was not distorted by the relatively mild autistic symptoms in part of the ASD sample, we separated participants with ASD who scored above the ADOS cutoff (7 or higher; High ADOS group) from those who did not meet the ADOS cutoff criterion (6 or lower; Low ADOS group). Consequently, we analyzed and compared three ASD groups with the TD group: the total ASD group, the High ADOS, and the Low ADOS group. There was a significant age difference between the total ADOS group (13.4), the High ADOS group (12.7), and the TD group (12.3), but not the Low ADOS group (13.8). There was no difference in receptive vocabulary test scores (The Peabody Picture Vocabulary Test, PPVT) between all groups (scores ranged from 103 to 107). Gender distribution in the total ADOS group (87 % boys) did not differ from the TD group (89 % boys), but the High ADOS (96 % boys) differed significantly from the Low ADOS group (81 % boys). The High ADOS group was therefore not only younger but also included more boys than girls compared to the Low ADOS group (see Table 1).

### Participants of Test–Retest Study

The test–retest reliability of the IDT was determined in a separate sample of 31 children (19 boys and 12 girls) with a mean age of 10.2 years (*SD* = 1.7), including 15 children with ASD (*M* = 11.41 years, *SD* = 0.73) and 16, slightly younger children with typical development (*M* = 9.11 years, *SD* = 1.62), recruited using the same procedures as in the main study. The ASD test–retest group included more boys (80 %) and fewer girls (20 %) than the TD test–retest group (44 % boys, 56 % girls). The estimated receptive vocabulary skills scores in the ASD (*M* = 101, *SD* = 11) and the TD group (*M* = 105, *SD* = 8.1) were similar. In the ASD group, SRS scores (*M* = 80.8, *SD* = 25.3, range 44–123) failed to confirm the ASD diagnosis of one participant. One participant from the TD group (*M* = 25.2, *SD* = 17.7, range 6–84) scored within the ASD range. Both these participants were excluded from the analyses. The test–retest time interval was 14–72 days with a mean retest interval of 45.2 days (14–72 days). There was no significant difference between test–retest time interval of ASD and TD participants.

### Procedure

After receiving informed consent from parents and participants (if 12 years or older), children and adolescents were asked to participate. Psychologists and graduate psychology students were trained to administer the IDT, using the extensive IDT manual (Backer van Ommeren et al. 2012b) and practicing, first with a supervisor, then with colleagues and finally with several typically developing test children. In the study all IDT administrations were videotaped, to monitor all performances and to score behaviors. The ADOS was administered by trained researchers. Testing the participants took place at their schools. All tests were administered by trained psychologists and master students, and took place at the participants’ schools. The study was approved by the ethics board of the school.

### Measures

Interactive Drawing Test (IDT; Backer van Ommeren et al. 2012a, b). Materials included a sheet of drawing paper (A3), pencils, and a camera to videotape the drawing process. Throughout the test, which lasted 10 min, the researcher drew according to specific instructions (see Backer van Ommeren et al. 2012a, b, for more details). The instructions to the participant were minimal, and included only one sentence at the beginning of the procedure: “We are going to draw together.” After this instruction, the researcher drew a single line on the paper and then turned the paper over (so it would face the participant) and pushed it across the table to the participant, to indicate that it was now his or her turn. Subsequently, the researcher and the participant took turns in adding elements to the drawing, using different colored pencils. From the start of the IDT administration, the researcher was instructed to refrain from changing the nature of the participant’s drawing when working together in his/her own subject matter. For instance, if the participant drew a tree, the researcher could add leaves to the tree.

Halfway through the test, the researcher was instructed to interfere with the participant’s drawing by adding three specific types of elements. These elements were designed to have a distinctive impact. First, the researcher added an *interfering* element that changed the nature of the participant’s drawing but fitted within the context (e.g., turning a figure of a child into a girl by adding a dress). Second, an *absurd* element was added, which always included adding two arms at an absurd location in the drawing (e.g., attached to an airplane). Third, the researcher added an element that had a *damaging* impact on the participant’s drawing object. This element always consisted of a bolt of lightning hitting an object drawn by the participant. After the participant had either accepted or rejected this last element, the researcher asked the participant if he/she thought the drawing was finished, or whether it needed another addition. The participant was allowed to make this addition.

#### Scoring

##### Number of Turns

To neutralize the influence of the number of turns on IDT scores, outcomes were based on the proportion of specific behaviors in relation to the total number of turns.

##### Reciprocal Turn Taking

We scored reciprocal turn taking by awarding points if the participant was active in turn taking by copying the researcher’s turn-taking behavior i.e., pushing and rotating the paper back to the researcher after his/her turn, so the drawing would face the researcher. Participants scored one point if they pushed the paper back to the researcher and two points if they also rotated the paper back. Reciprocal turn-taking behavior scores were computed as a proportion of the total number of turns, with higher scores reflecting more reciprocal turn-taking behavior.

##### Reciprocal Drawing

We scored reciprocal drawing by awarding one point each time the participant joined the researcher in drawing a mutual subject matter (e.g., the participant and researcher both contributed to the drawing of a tree). Total scores reflected the proportion of reciprocal drawing acts relative to the total number of turns, with higher scores indicating more reciprocal drawing.

##### Reciprocity in Other’s Initiative

We scored reciprocity in other’s initiative by awarding one point each time a participant contributed to an object initiated by the researcher. Total scores reflected the proportion of reciprocity in other’s initiative relative to the total number of turns. Higher scores indicated more reciprocal responding to the researcher’s initiatives.

##### Reciprocal Flexibility

We scored reciprocal flexibility by awarding points each time the participant responded to the researcher’s additions of the three specific types of elements. Participants scored one point if they incorporated the element (e.g., the participant draws a policeman behind the steering wheel in response to the researcher drawing a siren on the motorcar). Rejecting a contribution included scratching the addition away, changing it to fit the original concept, or ignoring it. The total score of reciprocal flexibility was based on the sum of the separate scores, with a maximum score of three points. Higher scores indicated more reciprocal flexibility.

##### Evaluation of Participation

After finishing the IDT, participants were asked to rate whether they had liked taking part in the drawing task on a 5-point scale (smileys) ranging from *very much* (5 points) to *not at all* (1 point).

The Autism Diagnostic Observation Schedule (ADOS; Lord et al. 2000, Dutch version; De Bildt et al. 2008) was used) assesses symptoms of autism across age, developmental level and language skills by observing social and communication behaviors. During a semi-structured observation, the ADOS interviewer offers playful activities (e.g., reading a story book) and topics of discussion (e.g., peer problems) to assess the socio-communicative abilities of the participant. Each of the participant’s behaviors is rated on a scale ranging from normal behavior (0) to clearly deviant and autistic behavior (2). An ADOS score of 7 or higher is indicative of an ASD. The ADOS has excellent internal consistency, inter-rater reliability, test–retest reliability, and discriminant validity (Lord et al. 2000; 2008; Molloy et al. 2011). We administered the ADOS Module 3 (used with verbally fluent children) and Module 4 (used with fluent adolescents and adults). A standardized continuous ADOS score is available (Gotham et al. 2009). However, Module 4, administered in 63 % of the ASD group, is not standardized, which is why we used the aggregated scores.

The Social Responsiveness Scale (SRS; Constantino and Gruber 2005, Dutch version Roeyers et al. 2011), measures the severity of autism spectrum symptoms as they occur in natural social settings, with a 65-item questionnaire completed by parent or teacher. Several studies found evidence for good test–retest reliability, interrater reliability, construct validity, convergent validity, (with the ADOS, ADI-R) and internal consistency of the SRS (Bolte et al. 2008; Wigham et al. 2012).

The Peabody Picture Vocabulary Test (PPVT; Dunn and Dunn 2004) is designed as a test of receptive vocabulary. The test consists of a series of pictures and is suitable for a wide age range (2–90 years). The participant has to match an orally given word to a picture. The reliability of the PPVT tested with split–split half and test–retest administration is excellent and the construct and content validity good (Bucik and Bucik 2003). The validity of the PPVT is evidenced by strong correlations between PPVT scores and overall intelligence (Bee and Boyd 2004; Bell et al. 2001).

## Results

### Evaluation of Participation

The average rating of participation in the IDT was 4.3, SD 0.7. Eighty-nine per cent of the participants liked participation “much” to “very much”, 11 % rated “indifferent” and only one participant rated it as “not very much”. No group differences or correlations were found between the participation ratings and any of the IDT measures. The mean total drawing time of all participants was less than 10 min. No differences were found in drawing time in the TD group 495 s. (*SD* 173 s.), the Low ADOS, 538 s. (*SD* = 205 s.) and the High ADOS group, 515 s. (*SD* = 159 s.).

### Reliability

#### Inter-rater Reliability

We assessed inter-rater reliability of the IDT scores by computing intraclass correlation coefficients (ICC) between IDT scores given by two blind, independent raters rating the performance of 20 participants (15 ASD and 5 TD) randomly taken from the main sample. Inter-rater reliabilities varied from .95 to 1.00 (1.00 for reciprocal turn taking, .99 for reciprocal drawing, .98 for reciprocity to other’s initiative, and .95 for reciprocal flexibility), indicating excellent levels of inter-rater reliability.

#### Test–Retest Reliability

We assessed test–retest reliability in the separate sample of 29 children. There was no significant difference between test–retest time intervals of ASD and TD participants. ICCs for all four IDT scores ranged from .47 to .70 (.70 for reciprocal turn taking, .70 for reciprocal drawing, .47 for reciprocity in other’s initiative, and .52 (*p* < .01) for reciprocal flexibility, indicating moderate to good test–retest reliability of the IDT measurements.

### Validity

#### Criterion-Related Validity

**Fig. 2:**
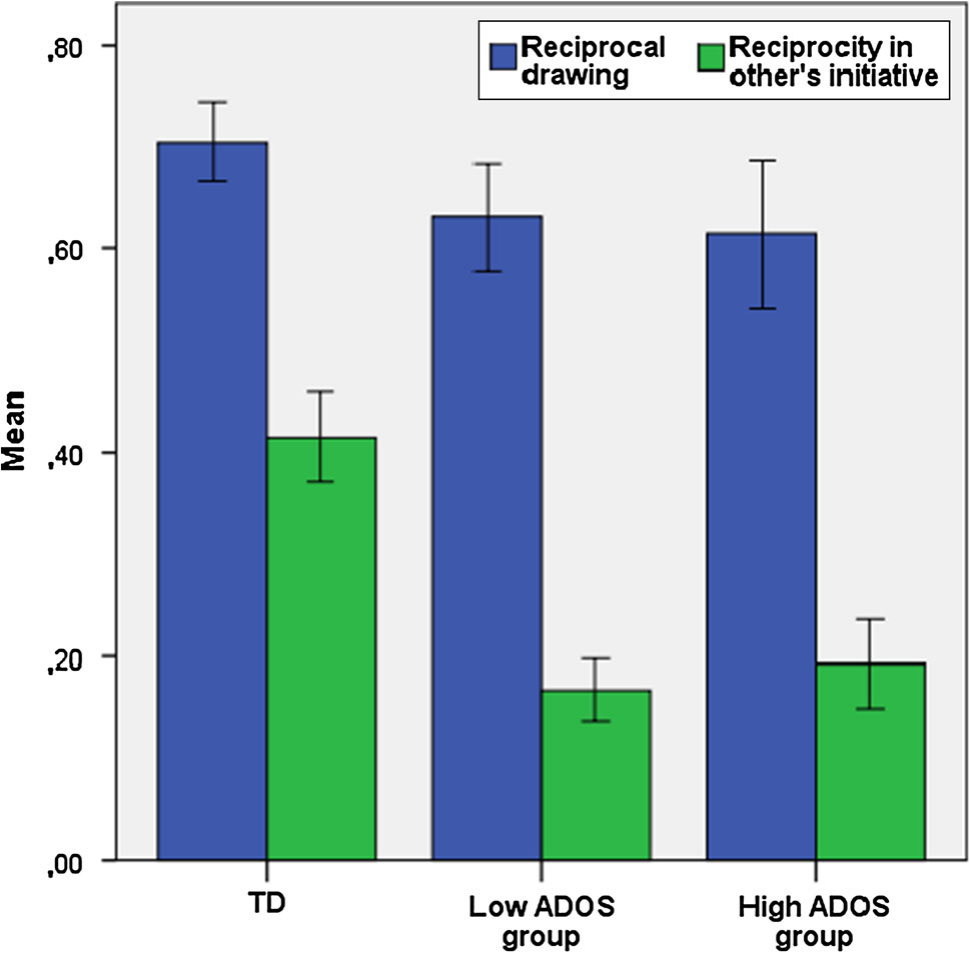
Percentage of reciprocal drawing and of reciprocity in other’s drawing initiatives in participants from TD, Low ADOS and High ADOS group

**Fig. 3:**
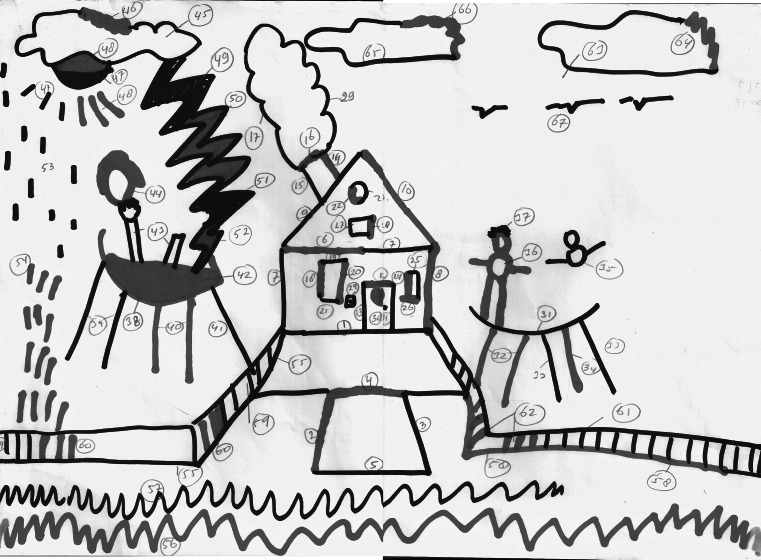
Example of a drawing by a 6 years old typically developing boy (*red marker*). This drawing shows strong reciprocal responding to the initiatives of the researcher. The boy joins the researcher from the start (drawing a house) by adding meaningful elements (Color figure online)

**Fig. 4:**
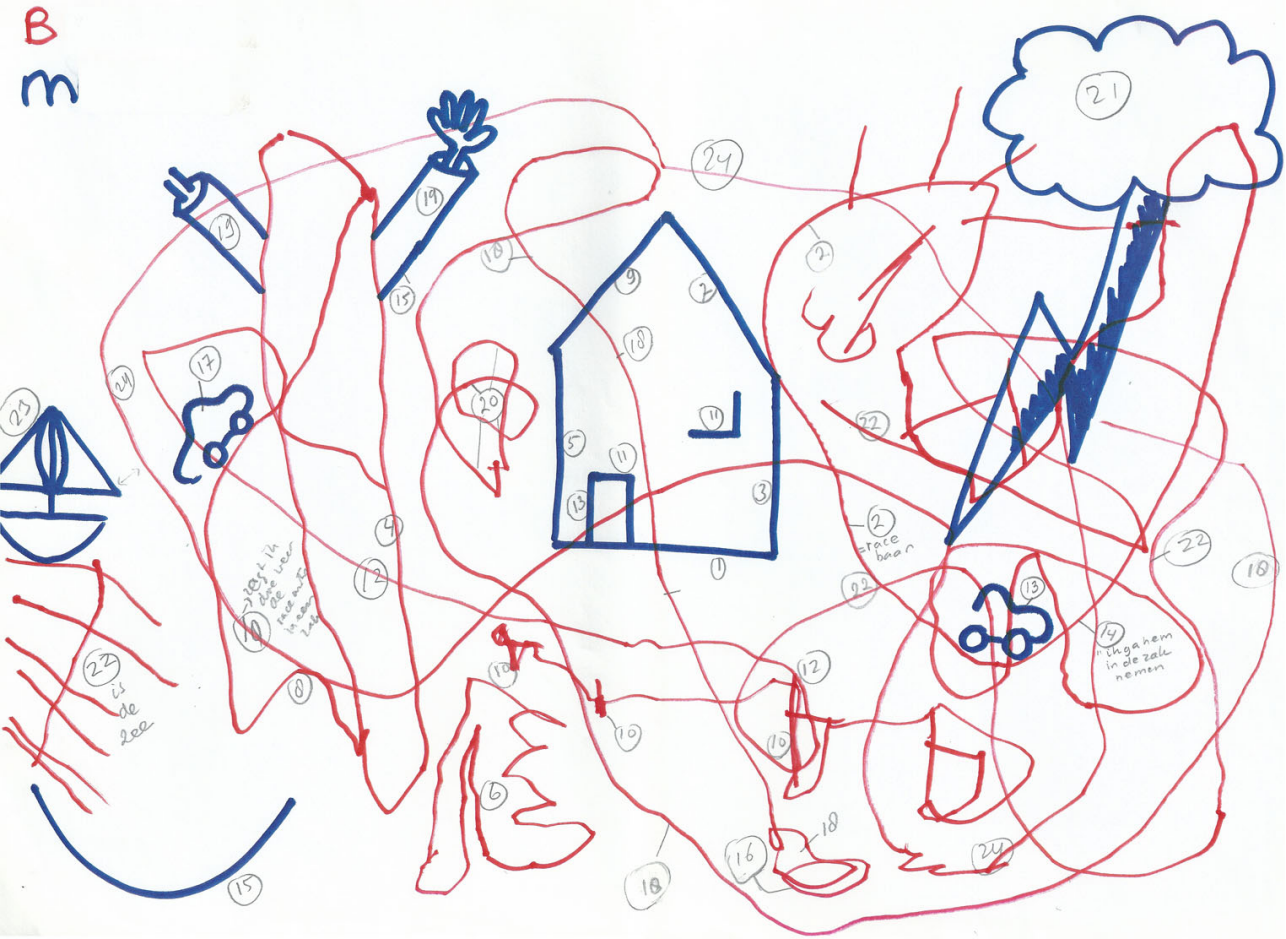
Example of a drawing by a 6 years old boy with ASD (*red marker*). This drawing shows the lack of reciprocal response to the initiatives of the researcher. The boy does not join the researcher at all and continues to draw his own objects (race circuits for motorcars) (Color figure online)

To test the criterion-related validity of the IDT, we conducted multiple analyses of variance with Group as a between factor, including two (TD vs. ASD) and three (TD vs. High ADOS and Low ADOS) levels, controlling for age. See Table 2 for means, standard deviations and effect sizes, and Figs. 2, 3 and 4 for illustrations of the key findings.

**Table 2:** IDT scores for the ASD High ADOS group, the ASD Low ADOS group, the total ASD group and the typically developing comparison group

IDT measures	High ADOS group (n = 51)		Low ADOS group (n = 80)		Total ASD group (n = 131)		Comparison group (n = 62)		Group differences	Effect sizes (η_p_^2^)		
										Total ASD versus comparison	High ADOS versus comparison	Low ADOS versus comparison
	M (SD)	Range	M (SD)	Range	M (SD)	Range	M (SD)	Range				
Number of turns	13.5 (3.2)	5–29	14.1 (4.7)	7–21	13.9 (4.2)	5–29	16.2 (4.8)	5–25	(High & Low) < C**	.08	.13	.07
Reciprocal turn taking	.81 (.67)	0–2	1.04 (.67).	0–2	.95 (.68)	0–2	1.48 (.54)	0–2	(High & Low) < C**	.13	.25	.10
Reciprocal drawing	.61 (.26)	0–1	.63 (.24)	0–1	.62 (.24)	0–1	.71 (.15)	.27–1	(High & Low) < C*	.03	.05	.03
Reciprocity in other’s initiative	.19 (.16)	0–.54	.17 (.14)	0–.73	.18 (.14)	0–.73	.41 (.17)	0–.82	(High & Low) < C**	.36	.33	.40
Reciprocal flexibility	1.7 (1.1)	0–3	1.9 (.91)	0–3	1.8 (.97)	0–3	2.5 (.60)	1–3	(High & Low) < C**	.13	.21	.12

* *p* < .05; ** *p* < .001

##### Number of Turns

TD participants took more turns than ASD participants, indicating that there were more reciprocal interactions between TD participants and the researcher than between ASD participants and the researcher (*F*_(1,190)_ = 16.65, *p* < .001, *η*_*p*_^2^ = .08). TD participants also took more turns together with the researcher, also when compared separately to the Low ADOS (*F*_(1,139)_ = 10.22, *p* < .05, *η*_*p*_^2^ = .07) and the High ADOS group (*F*_(1,110)_ = 15.72, *p* < .001, *η*_*p*_^2^ = .13). The number of turns increased with age, but only in the TD group (*r* = .33, *p* < .01).

##### Reciprocal Turn Taking

TD participants showed more reciprocal turn taking compared to the total ASD group (*F*_(1,181)_ = 26.44, *p* < .001, *η*_*p*_^2^ = .13), and compared to the Low ADOS group (*F*_(1,130)_ = 13.64 *p* < .001, *η*_*p*_^2^ = .10) and the High ADOS group (*F*_(1,101)_ = 33. 62, *p* < .001, *η*_*p*_^2^ = .25).

##### Reciprocal Drawing

TD participants joined the researcher in drawing a mutual subject matter more frequently compared to ASD participants (*F*_(1,190)_ = 5.57, *p* < .05, *η*_*p*_^2^ = .03) and compared to the Low ADOS group (*F*_(1,139_ = 4.30, *p* < .05, *η*_*p*_^2^ = .03) and the High ADOS group (*F*_(1,10_ = 5.37, *p* < .05, *η*_*p*_^2^ = .05).

##### Reciprocity in Other’s Initiative

TD participants contributed to an object initiated by the researcher more frequently compared to the total ASD group (*F*_(1,190)_ = 105.34, *p* < .001, *η*_*p*_^2^ = .36) and compared to the Low ADOS (*F*_(1,139)_ = 94.29, *p* < .001, *η*_*p*_^2^ = .40), and the High ADOS group (*F*_(1,110)_ = 53.87, *p* < .001, *η*_*p*_^2^ = .33). The limitations in reciprocating to the initiative of the researcher can be illustrated by inspecting Figs. 3 and 4. The house figure in the middle is a drawing initiative of the researcher. It is evident that the participant with autism did not reciprocate to that initiative, while the typically developing participant showed clear reciprocal behavior. This distinction is representative for our main findings, see Fig. 2.

##### Reciprocal Flexibility

TD participants accepted all of the researcher’s contributions more frequently compared to the total ASD group (*F*_(1,190)_ = 27.78 *p* < .001, *η*_*p*_^2^ = .13), and compared to the Low ADOS group (*F*_(1,139)_ = 18.73, *p* < .001, *η*_*p*_^2^ = .12), and the High ADOS group (*F*_(1,110)_ = 28.29, *p* < .001, *η*_*p*_^2^ = .21).

None of the IDT score comparisons between the Low and the High ADOS group yielded a significant difference (*F*_(1,128)_ = 3.13, *ns* for reciprocal turn taking, *F*_(1,128)_ = .14, *ns* for reciprocal drawing, *F*_(1,128)_ = 1.56, *ns* for reciprocity in other’s initiative, and *F*_(1,128)_ = 1.11, *ns* for reciprocal flexibility).

#### Convergent Validity

To assess the convergent validity of the IDT, we tested the relation of IDT scores with the severity of ASD symptoms as reflected by SRS and ADOS scores, see Table 3. Based on all participants combined SRS total scores were significantly correlated with all IDT outcomes, indicating that higher IDT scores corresponded with lower severity of autism symptoms according to the SRS. ADOS scores were available from the ASD group only. A modest negative correlation was found between ADOS total score and reciprocal turn taking, showing less turn taking in more severe autism scores on the ADOS. No other correlations were found, and, surprisingly, scores on the ADOS reciprocal social interaction subtotal were not correlated with any of the IDT outcomes. When we analyzed the IDT scores of the ASD low ADOS and the ASD high ADOS groups separately, no significant correlations with the IDT were found.

**Table 3:** Correlations between SRS and ADOS total scores and ADOS sub module reciprocal social interaction of the total ASD group, and the Low and High ADOS group separately

	Reciprocal turn taking	Reciprocal drawing	Reciprocity in others initiative	Reciprocal flexibility
ASD and TD group combined (n = 193)				
SRS total score	−.25**	−.17*	−.48**	−.26**
ASD group (n = 131)				
SRS total score	.05	−.09	−.07	−.06
ADOS total score	−.18*	−.01	−.08	−.13
ADOS Reciprocal social interaction	−.13	.04	.04	−.13
Low ADOS group (n = 80)				
SRS total score	.13	−.18	−.07	−.04
ADOS total score	−.21	.09	−.02	.06
ADOS Reciprocal social. Interaction	−.08	.14	−.14	.03
High ADOS group (n = 51)				
SRS total score	.10	.13	−.09	−.15
ADOS total score	.04	.01	−.06	−.17
Reciprocal social interaction	.09	.13	−.09	−.15

** *p* < .01; * *p* < .05

#### Divergent Validity

Correlations with PPVT scores of all participants or separately for TD and ASD participants were non-significant and ranged from *r* = −.04 to *r* = .11, indicating that IDT scores were independent of receptive vocabulary. To analyze gender differences, we performed multiple analyses of variance, with Gender as a between factor, controlling for age. We found no significant gender differences in the IDT scores of all participants (*F*_(1,182_ = .34 *ns* for reciprocal turn taking, *F*_(1,190_ = .03, *p* = *.8*6 *ns* for reciprocal drawing, *F*_(1,190_ = .81 *ns* for reciprocity in other’s initiative, and *F*_(1,190_ = .19, *ns* for reciprocal flexibility). Separate analyses of TD and ASD group scores revealed no gender differences either.

## Discussion

We found strong support for the reliability and validity of the IDT. The IDT was highly reliable, as demonstrated by the excellent inter-rater reliabilities. Test–retest reliability was moderate to good, even though the IDT is designed to elicit *spontaneous* reciprocal behavior, and a small learning curve could be expected. However, this quality of the IDT is necessary to reflect real life behavior without relying on rules or explicit instructions (Channon et al. 2001). Criterion validity of the IDT was supported by clearly distinct patterns of outcomes in typically developing participants and participants with ASD. Compared to the TD group, ASD participants scored considerably lower on all four IDT measures (reciprocal turn taking, reciprocal drawing, reciprocity in other’s initiative, and reciprocal flexibility). Effect sizes were medium (in reciprocal drawing) to very large (as expected, in reciprocity in other’s initiative). These findings confirm the pilot study (Backer van Ommeren et al. 2012a, b).

The most striking difference between the ASD and the TD group was the ability to reciprocate *another person’s initiative*, making this the most sensitive outcome of the IDT for autism. The ability to reciprocate in general, irrespective of who initiated the drawing interaction, was only slightly lower in the ASD than the TD group, with both groups showing reciprocity in the majority of drawing interactions (see Table 2). This indicates that ASD participants were not incapable of showing reciprocal behavior. However, they *primarily* showed this behavior when *they* *themselves* had initiated the drawing elements, and were in control of the topic of the interaction. In contrast, strong limitations were found in their ability to show reciprocity when the researcher controlled the drawing topic. This finding shows how the level of control exerted by individuals with autism influences their reciprocal behavior.

Interestingly, while we found a high effect size of the group difference on reciprocity in other’s initiative, test re-test reliability on this domain were lowest. We think that this can be explained based on the responses of the TD children, who showed particularly low test–retest reliability on reciprocity in other’s initiative. This could be explained by the fact that they were more familiar and comfortable with the researcher during the retest, and dared to show more initiative by drawing their own objects besides following the initiative of the researcher. In the ASD children we did not found this effect in the retest, indicating that they still prefer to draw their own objects.

The limited flexibility of participants with ASD in response to specific additions of the researcher to participant-initiated elements of the drawing again underlines the importance of them being in charge of the interaction. For instance, when the participant drew a car, and the researcher changed the car into a fire engine, the majority of typically developing participants embraced the addition and continued to contribute to the fire engine. In contrast, the ASD participants generally ignored the addition, scratched it away, or started drawing elsewhere. On a more basic level, ASD participants also showed a lower frequency of reciprocal turn taking compared to the typically developing group (i.e., pushing and rotating the paper back to face the researcher). This indicates elementary limitations in reciprocal behavior, such as showing behavior that is similar to the other person’s actions.

Convergent validity was tested by analyzing the relation of IDT scores with the severity of ASD symptoms as rated by the ADOS and SRS. Within the ASD sample, correlations between IDT and ADOS or SRS scores, were absent or low. Thus, although the IDT appeared to be highly sensitive to differences in reciprocity of children and adolescents with and without ASD, the IDT does not seem to be sensitive to the severity of autism among individuals with an ASD diagnosis as measured by the ADOS or the SRS.

The validity of the ADOS has recently been re-established (Hus and Lord 2014), and the ADOS provides a reliable proxy on reciprocal behavior, based on a broad domain of outcomes (Lord et al. 2000). In contrast, the IDT measures the frequency and the quality of explicitly defined reciprocal behavior. This more specific approach focused only on reciprocal behavior may explain the poor correspondence between the IDT and the ADOS, despite the convergence of the IDT with levels of ASD severity measured by parental reported autistic traits assessed by the SRS. However, it should be noted that no ADOS scores were available for the typically developing participants, thus restricting its score range.

The divergent validity of the IDT was good. We confirmed that IDT scores were independent of verbal intelligence and age, except for the number of turns taken in the TD group, which increased with age. Older TD participants may be more aware of the interactive nature of a mutual drawing. This had no effect on our main findings. All analyses were controlled for age, and reciprocal behavior was scored in proportion to the number of turns taken. Gender did not affect IDT performances. However, the small number of girls in our sample warrants caution in generalizing these results. Earlier studies indicated that social impairments are more subtle in girls than in boys with ASD (Dworzynski et al. 2012; Kothari et al. 2013).

The IDT targets reciprocal nonverbal behavior. Its independence of verbal abilities is an important benefit for the assessment in cognitively able individuals with autism. The implicit and nonverbal nature of the IDT allows little opportunity for cognitive compensation, a strategy often applied by high-functioning individuals with autism to circumvent limited intuitive social skills (Scheeren et al. 2013). Its independence of verbal abilities makes the IDT potentially suitable for cognitively delayed or otherwise verbally impaired individuals with ASD.

Several limitations of the findings of this study should be noted. The sample only comprised school-aged children and adolescents with average or above average receptive vocabulary skills. Therefore, our findings cannot be generalized to adults, or individuals with cognitive impairments or younger children. A larger selection of females with ASD is needed for a more exact assessment of gender differences and more thorough IQ measures, including indications of non-verbal abilities, would have provided a better indication of cognitive abilities. The IDT relies on an interaction with an adult. Peer interactions, which may be even more difficult for children with ASD, were not assessed (Bauminger-Zviely et al. 2013). The reliability of the IDT depends on the ability of the researcher, who interacts, observes and initiates reciprocal behavior simultaneously. While training has been shown to result in reliable administration and we strive for objective measures, the process or reciprocity will include subjective elements. Finally, despite their clinical diagnosis, which was based on an extensive clinical procedure, independent from and prior to the current study, a large part of the ASD sample did not receive ADOS scores in the clinical range. This may be due to features or the high functioning sample, but could also be linked to the limited clinical experience in some of the ADOS administrators. We dealt with this issue by dividing the sample in a low and high ADOS group. Future studies in new samples will be needed to confirm the generalization of the current findings results.

Despite these limitations, the results of this study suggest that the IDT is a reliable and valid instrument for measuring the dynamic nature of reciprocal interactions in children and adolescents with normal intellectual abilities, as part of the procedures needed to establish an ASD diagnosis. Finally, the measures of reciprocity obtained with the IDT might be used in studies targeting individual differences in reciprocity, both in children with and without ASD.
